# Live-cell imaging of endogenous Egr1 mRNA using an MPBS knock-in mouse model

**DOI:** 10.1016/j.isci.2026.115955

**Published:** 2026-04-30

**Authors:** Hyerim Ahn, Hyeonjeong Jeong, Dong Wook Kim, Jae Youn Shim, Hye Yoon Park

**Affiliations:** 1 Department of Electrical and Computer Engineering, College of Science and Engineering, University of Minnesota, Minneapolis, MN 55455, USA; 2 Department of Physics and Astronomy, College of Natural Sciences, Seoul National University, Seoul 08826, Republic of Korea; 3 Department of Biomedical Engineering, College of Science and Engineering, University of Minnesota, Minneapolis, MN 55455, USA; 4 Graduate Program in Neuroscience, University of Minnesota, Minneapolis, MN 55455, USA

**Keywords:** molecular biology, molecular mechanism of gene regulation

## Abstract

Real-time imaging of endogenous mRNA is essential for understanding the dynamics of gene regulation, yet appropriate animal models have been limited. We developed an Egr1-MPBS knock-in mouse line in which all endogenous Egr1 transcripts are tagged with 12 tandem MS2 and PP7 binding site (MPBS) pairs, enabling direct visualization of single mRNA molecules under physiological conditions. To establish proof of principle, we derived mouse embryonic fibroblast (MEF) lines from this model and performed live-cell imaging of fluorescently labeled Egr1 transcripts. We found that the insertion of the MPBS cassette did not alter endogenous mRNA stability or expression dynamics. This system allowed us to observe Egr1 transcriptional bursting and quantitatively track individual mRNA molecules as they diffused within the nucleus and cytoplasm. By integrating technological innovation with mechanistic insight, the Egr1-MPBS mouse establishes a broadly applicable platform for quantitative studies of gene expression and RNA trafficking at single-molecule resolution in living systems.

## Highlights

• New Egr1-MPBS mouse line for real-time imaging of endogenous Egr1 transcripts
• MPBS tagging preserves endogenous mRNA stability and native expression dynamics
• Quantitative tracking of transcriptional bursting and mRNA spatial diffusion
• A versatile platform for single-molecule studies of gene regulation in living systems

## Graphical abstract

**Figure:**
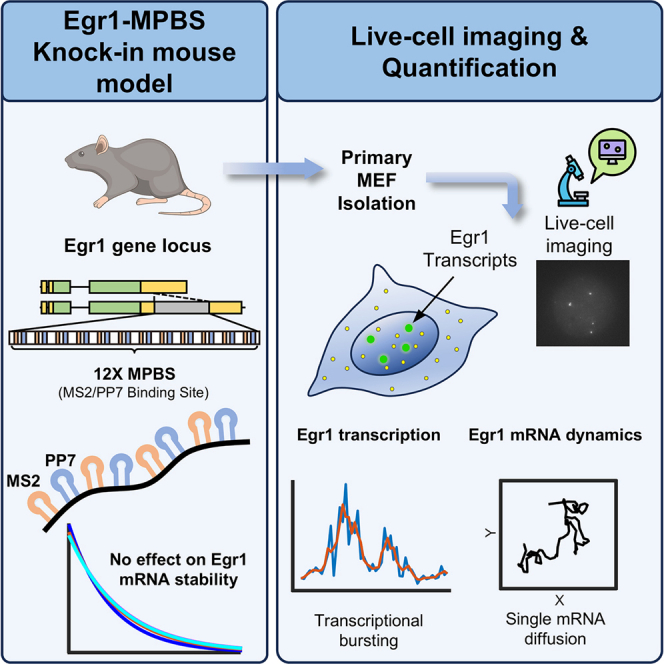

## Introduction

Early growth response 1 (Egr1), also known as NGFI-A, zif268, or Krox-24, is an immediate-early gene (IEG) encoding a zinc-finger transcription factor involved in diverse cellular processes. It was independently identified in the late 1980s as a nerve growth factor (NGF)-inducible gene (NGFI-A) in neuron-like PC12 cells^1^ and as a 12-*O*-tetradecanoylphorbol-13-acetate (TPA)-inducible transcript (TIS8) in fibroblasts.^2^ Since then, Egr1 has been shown to be expressed in a wide range of tissues and cell types, including fibroblasts, epithelial cells, endothelial cells, and immune cells, where it regulates proliferation, differentiation, apoptosis, and stress responses.^3^^,^^4^^,^^5^ This widespread and stimulus-inducible expression pattern highlights its role as a general regulator of activity-dependent transcription across broad biological contexts.

In recent years, Egr1 has been studied most extensively in the field of neuroscience, where its rapid and transient induction in neurons has been linked to synaptic plasticity and memory. Genetic and physiological studies have shown that Egr1 is required for the induction and maintenance of long-term potentiation (LTP), particularly in its late phases, and that the disruption of Egr1 impairs memory consolidation and reconsolidation.^6^^,^^7^^,^^8^^,^^9^ These findings have established Egr1 as a central molecular marker of neuronal activity and memory-related plasticity.

To monitor Egr1 expression, several transgenic mouse models have been developed, in which the Egr1 promoter drives reporter genes such as LacZ,^10^ luciferase,^11^ or fluorescent proteins such as EGFP.^12^^,^^13^ These approaches have provided valuable insights into the regulation of Egr1 *in vivo*. However, promoter-driven reporter systems have inherent limitations, as reporter protein expression requires transcription, translation, and protein maturation, resulting in a delay of a few hours after the initial transcriptional event. In addition, the long half-life of reporter proteins, while useful for cumulative readouts, can obscure the transient and dynamic nature of endogenous Egr1 regulation, and reporter localization may not always coincide with native Egr1 protein distribution.^14^ Thus, there remains a need for methods that enable real-time monitoring of Egr1 expression directly at its endogenous locus.

To achieve such real-time visualization of endogenous mRNA, bacteriophage-derived stem-loop systems have been successfully applied in living cells.^15^^,^^16^^,^^17^ In these approaches, tandem arrays of RNA stem-loops such as MS2 or PP7 binding sites (MBS or PBS) are inserted into the 3′ or 5′ untranslated region (UTR) of a gene of interest. Fluorescent labeling is achieved by expressing MS2 or PP7 capsid proteins (MCP or PCP) fused to fluorescent proteins, which bind to their corresponding RNA-binding sites during transcription.^18^^,^^19^ Knockin (KI) mouse models using these systems have provided powerful tools to study the dynamics of endogenous mRNAs *in vivo*. For example, β-actin (Actb) mRNA dynamics have been monitored using Actb-MBS KI mice,^20^^,^^21^^,^^22^^,^^23^^,^^24^^,^^25^^,^^26^^,^^27^ and the Activity-regulated cytoskeleton-associated protein (Arc) mRNA dynamics have been monitored using Arc-PBS KI mice.^23^^,^^27^^,^^28^^,^^29^^,^^30^^,^^31^^,^^32^ These mouse models have enabled real-time and quantitative analyses of transcriptional bursting, intracellular transport, and localization of gene-specific mRNAs at single-molecule resolution. Moreover, the orthogonality of the MS2 and PP7 systems enables dual-color imaging of distinct mRNAs without cross-reactivity.^33^

Building on these advances, we generated an Egr1-MPBS KI mouse model, in which 12 pairs of MS2 and PP7 binding sites (MPBSs) were inserted into the 3′UTR of the endogenous Egr1 gene. This alternating MPBS configuration was strategically selected to enable orthogonal recruitment of different RNA-binding proteins, providing a modular and extensible platform for achieving robust signal detection with minimal background^34^^,^^35^ and allowing for the potential integration of functional protein assemblies.^36^ As proof of principle, we derived immortalized mouse embryonic fibroblast (MEF) lines from this model and confirmed that the insertion of the MPBS cassette did not perturb endogenous mRNA stability or expression kinetics. This approach enabled us to directly compare the performance of MS2 and PP7 tagging strategies under the identical genetic and experimental conditions, allowing robust evaluation of their effects on Egr1 transcriptional dynamics. Live-cell imaging of fluorescently labeled transcripts further revealed the distinctive kinetics of Egr1 transcriptional bursting and enabled direct measurement of Egr1 mRNA diffusion coefficients in both the nucleus and cytoplasm. Together, these findings establish the Egr1-MPBS mouse as a versatile platform for real-time, single-molecule studies of gene expression and RNA dynamics under native regulatory control.

## Results

### Generation of the Egr1-MPBS knock-in mouse model

**Figure 1:**
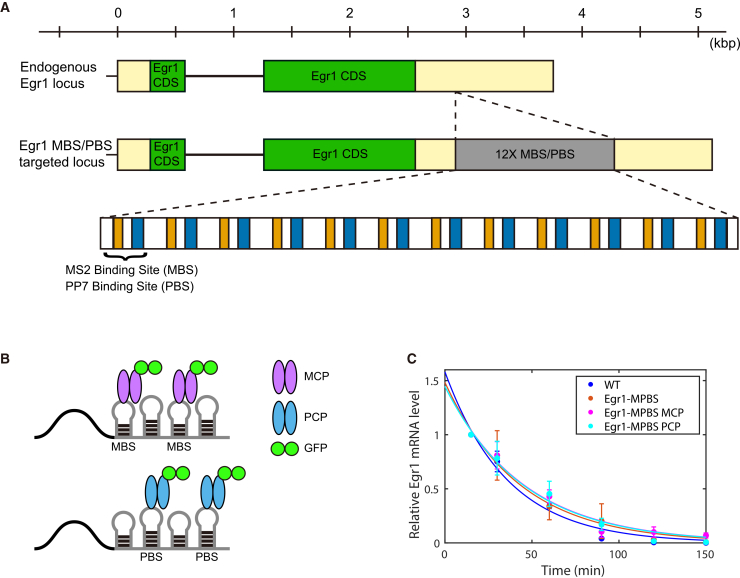
Generation of the Egr1-MPBS mouse model and the GFP labeling system (A) Schematic of the endogenous Egr1 locus and the final targeted locus. Green boxes indicate the Egr1 coding sequence; yellow boxes, untranslated regions (UTRs); gray box, the 12× MPBS cassette composed of orange boxes (MBS) and blue boxes (PBS); black lines, introns. (B) Schematic of the GFP labeling system using the MBS or PBS inserts in combination with MCP-GFP or PCP-GFP constructs. The stem-loop sequences in the 3′UTR of Egr1 transcripts bind to their respective coat proteins (MCP-GFP or PCP-GFP), allowing real-time visualization of Egr1 transcription dynamics in live cells. (C) Measurement of Egr1 mRNA half-life in WT and Egr1-MPBS MEFs expressing MCP-GFP or PCP-GFP. Error bars indicate mean ± SD from three independent experiments (triplicates, outliers removed). Decay curves were fitted with a single-exponential function (solid lines). Two-way ANOVA with replication showed no statistically significant differences among groups or in the group × time interaction (*p* > 0.05).

The Egr1 gene spans 3,750 base pairs (bp) on mouse chromosome 18 and consists of two exons encoding a 533-amino acid protein. To enable the single-molecule imaging of Egr1 mRNA in live cells, we generated an Egr1-MPBS KI mouse model by inserting 12 tandem pairs of MBS and PBS into the 3′UTR of the Egr1 gene (Figure 1A). To avoid disrupting evolutionarily conserved *cis*-regulatory elements, we conducted a multi-species sequence alignment of the Egr1 locus and identified a less conserved region located 347 nucleotides downstream of the stop codon. The MPBS cassette was inserted at this site using CRISPR-Cas9 genome editing in the C57BL/6N genetic background. Out of 85 pups, two F0 founders were identified as successful knock-ins by PCR-based genotyping. Germline transmission was confirmed in the F1 generation, from which three independent Egr1-MPBS carriers were obtained. The KI mice were bred to homozygosity, and we have not found any overt phenotypes in the homozygous Egr1-MPBS mouse line.

To characterize the Egr1-MPBS KI line, we cultured MEFs and immortalized them by transfection with the SV40 large T antigen. For live-cell imaging of Egr1 mRNA, we infected MEFs with lentiviral vectors expressing tandem-dimer fusions tdMCP-tdGFP or tdPCP-tdGFP (hereafter referred to as MCP-GFP and PCP-GFP), which bind to the MS2 or PP7 stem-loop sequences, respectively (Figure 1B). The stability of Egr1 mRNA was assessed in MEFs derived from wild-type (WT) and Egr1-MPBS mice, with and without MCP-GFP or PCP-GFP expression. Egr1 mRNA expression was induced by serum stimulation, and transcription was subsequently inhibited at various time points by treatment with 5,6-dichlorobenzimidazole 1-β-D-ribofuranoside (DRB), a transcriptional elongation inhibitor. The decay of Egr1 transcripts following transcriptional blockade was quantified by quantitative real-time PCR (qRT-PCR) (Figure 1C). Fitting the relative mRNA levels in each MEF line to a single exponential decay function yielded average half-lives (*t*_1/2_) of 25 ± 2 min (mean ± SD) in WT MEFs, 29 ± 6 min in Egr1-MPBS MEFs, 32 ± 3 min in Egr1-MPBS MEFs expressing MCP-GFP (Egr1-MPBS × MCP), and 31 ± 4 min in Egr1-MPBS MEFs expressing PCP-GFP (Egr1-MPBS × PCP). These half-life values were not statistically different from one another (one-way ANOVA, *p* > 0.05). In addition, two-way ANOVA with replication showed no statistically significant differences among samples or in the interaction between sample group and time (*p* > 0.05). These results suggest that the insertion of the MPBS cassette and labeling with MCP-GFP or PCP-GFP had no detectable effect on Egr1 mRNA stability.

### smFISH validation of MPBS tagging of endogenous Egr1 mRNAs

**Figure 2:**
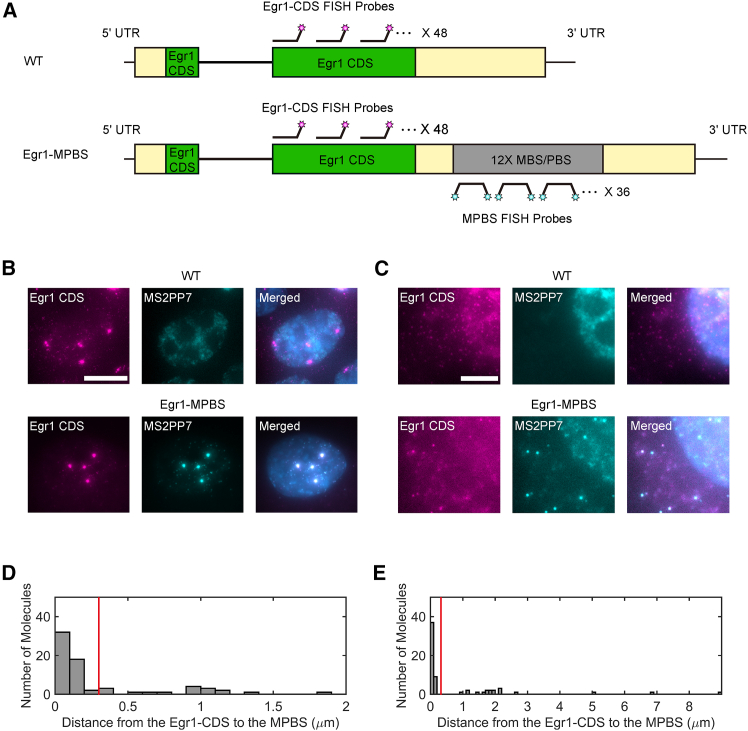
The MPBS-tagging system enables the visualization of Egr1 transcription and single mRNA molecules (A) Schematic of the smFISH strategy using probes targeting the Egr1 coding sequence (CDS) and the MPBS linker (MS2-PP7 stem-loop linker sequences). (B) Detection of transcription sites in MEFs from WT (top) and Egr1-MPBS (bottom) mice using CDS and MPBS probes. Scale bars, 10 μm. (C) Detection of single mRNA molecules in WT (top) and Egr1-MPBS (bottom) MEFs under steady-state conditions. Scale bars, 5 μm. (D and E) Histogram of nearest neighbor distances between CDS and MPBS FISH signals in the nucleus (D) and cytoplasm (E). A co-localization threshold of 300 nm was applied, as indicated by the red vertical lines.

We next examined whether the inserted MPBS sequences are faithfully transcribed and incorporated into Egr1 mRNAs in MEFs. We performed two-color single-molecule fluorescence *in situ* hybridization (smFISH) using probes targeting the Egr1 coding sequence (CDS probes) and probes specific to the linker regions within the MPBS cassette (MPBS probes) (Figure 2A). A mixture of three probes recognizing the 12× linker repeats was used for the detection of the inserted sequences (Table S1). Both WT and Egr1-MPBS MEFs exhibited Egr1 mRNA signals with the CDS probe, whereas MPBS probe signals were detected exclusively in Egr1-MPBS MEFs, indicating that the knock-in cassette is transcribed as part of endogenous Egr1 mRNAs and is specifically recognized by MPBS probes (Figures 2B and 2C). Under steady-state conditions, some MEFs exhibited active transcription sites in the nucleus (Figure 2B), while others showed individual Egr1 mRNA molecules in the nucleus, cytoplasm, or both (Figure 2C). Because MEFs are typically tetraploid, up to four Egr1 transcription sites were observed per cell.

To further evaluate transcript tagging, we quantified colocalization between CDS and MPBS signals. For each CDS-positive spot, the distance to the nearest MPBS spot was measured in the nucleus and cytoplasm (Figures 2D and 2E). Colocalization was defined as signals within 300 nm, based on the resolution limit of the microscopy system. Using this criterion, approximately 75% of nuclear and 72% of cytoplasmic mRNA signals were colocalized. These results demonstrate that the MPBS cassette is faithfully transcribed into endogenous Egr1 mRNAs and enables their reliable detection.

Furthermore, to directly validate the specificity and efficiency of the MPBS tagging system for protein-based visualization, we performed combined immunofluorescence (IF) for GFP with smFISH targeting the Egr1 CDS in MEFs expressing MCP-GFP and PCP-GFP. We observed a high degree of colocalization between the GFP-labeled puncta and Egr1 mRNA signals, whereas no significant colocalization was detected with Fos CDS probe used as a negative control (Figure S1). These findings collectively confirm that the GFP-labeled MPBS complexes specifically and accurately mark endogenous Egr1 transcripts, validating the robustness of our dual-labeling platform for real-time imaging.

### MPBS labeling does not disrupt endogenous Egr1 mRNA expression

**Figure 3:**
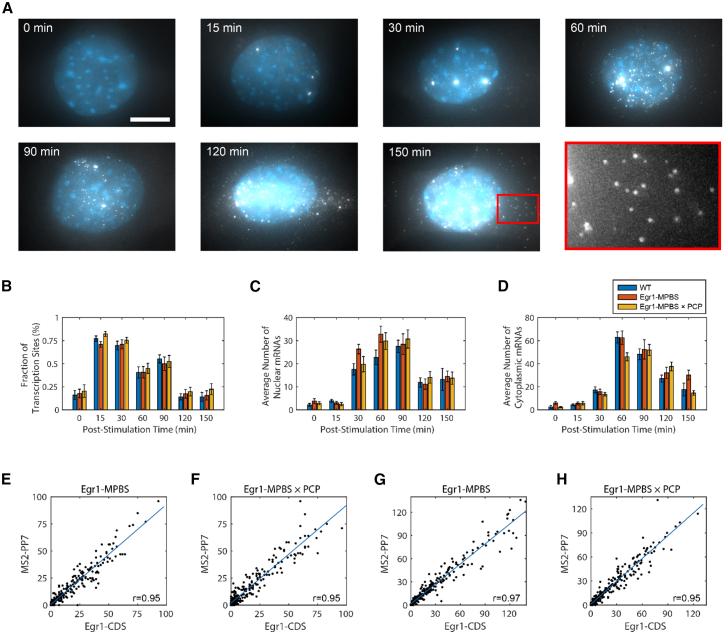
The MPBS labeling system does not affect endogenous Egr1 transcriptional dynamics or mRNA stability (A) Representative smFISH images of Egr1-MPBS MEFs. Cells were serum-stimulated and fixed at the indicated time points, followed by hybridization with Egr1 CDS probes (gray). Nuclei were stained with DAPI (blue). The high-magnification inset (red box) from the 150 min time point demonstrates that individual cytoplasmic mRNA spots are well-resolved. Scale bars, 10 μm. (B) Quantification of the average fraction of Egr1 transcription sites per nucleus, normalized to the maximum of four sites per cell. No statistically significant differences were detected among WT, Egr1-MPBS, and Egr1-MPBS × PCP groups across all time points (one-way ANOVA; *p* > 0.05). (C) Average number of nuclear Egr1 mRNA molecules per cell in WT, Egr1-MPBS, and Egr1-MPBS × PCP MEFs. Nuclear mRNA levels were comparable across groups (one-way ANOVA; *p* > 0.05). (D) Average number of cytoplasmic Egr1 mRNA molecules per cell. One-way ANOVA revealed no statistically significant differences across most time points (*p* > 0.05), except at 60 and 150 min, where modest differences were observed (0.005 < *p* < 0.05). (E–H) Correlation analysis of mRNA counts detected with CDS versus MPBS probes in the nucleus (E and F) and cytoplasm (G and H) of Egr1-MPBS and Egr1-MPBS × PCP MEFs. Strong Pearson’s correlation coefficients (r) confirm that MPBS labeling does not impair detection efficiency or nuclear export (*n* = 251 for Egr1-MPBS nuclei; *n* = 240 for Egr1-MPBS × PCP nuclei; *n* = 255 for Egr1-MPBS cytoplasm; *n* = 240 for Egr1-MPBS × PCP cytoplasm). Sample sizes for each time point were as follows: WT (*n* = 28, 56, 38, 37, 33, 30, 30), Egr1-MPBS (*n* = 31, 43, 44, 38, 27, 33, 35), and Egr1 PCP-GFP (*n* = 27, 34, 51, 40, 30, 29, 29). Error bars represent SEM.

We next examined Egr1 transcriptional kinetics following serum stimulation. WT, Egr1-MPBS, and Egr1-MPBS × PCP MEFs were serum-starved overnight, stimulated with 15% fetal bovine serum (FBS), and fixed at multiple time points (0, 15, 30, 60, 90, 120, and 150 min) for smFISH analysis (Figure 3A). We first quantified the fraction of active Egr1 alleles, based on the number of transcription sites observed per cell (up to four per nucleus). The proportion of alleles engaged in transcription increased sharply at 15 min after serum stimulation and then gradually declined over time, with no major differences among the three cell lines (Figure 3B).

Next, we examined the temporal distribution of Egr1 mRNAs in the nucleus and cytoplasm. Egr1 transcripts were scarcely detected before stimulation or at 15 min. Nuclear mRNAs began to accumulate at 30 min, peaked around 60–90 min, and decreased thereafter (Figure 3C). Cytoplasmic mRNAs first appeared at 30 min, reached maximal levels at 60 min, and then gradually declined (Figure 3D). One-way ANOVA revealed no statistically significant difference among the three cell lines at most time points (*p* > 0.05), with the exception of cytoplasmic mRNA at 60 and 150 min, where modest differences were detected (0.005 < *p* < 0.05). However, these differences were small, not consistently observed across experiments, and did not alter the overall temporal profile. The transcriptional kinetics were highly similar among all cell lines, indicating that MPBS labeling has little impact on Egr1 mRNA expression or subcellular distribution.

To further confirm labeling fidelity, we compared mRNA counts detected by CDS and MPBS probes in individual cells. Strong Pearson’s correlations were observed in both the nucleus (Egr1-MPBS: r = 0.95; Egr1-MPBS × PCP: r = 0.95) (Figures 3E and 3F) and cytoplasm (Egr1-MPBS: r = 0.97; Egr1-MPBS × PCP: r = 0.95) (Figures 3G and 3H). These results demonstrate that MPBS signals faithfully recapitulate endogenous Egr1 mRNA levels. Notably, unlike the MS2-MCP system in yeast, where binding site arrays were shown to generate aberrant 3′ mRNA decay fragments,^37^ such artifacts were not detected in our Egr1-MPBS system. Together, these results suggest that the MPBS labeling system provides a reliable readout of endogenous Egr1 mRNA expression without detectable perturbation of transcription, mRNA processing, nuclear export, and cytoplasmic localization.

### Live-cell imaging of Egr1 transcriptional dynamics

**Figure 4:**
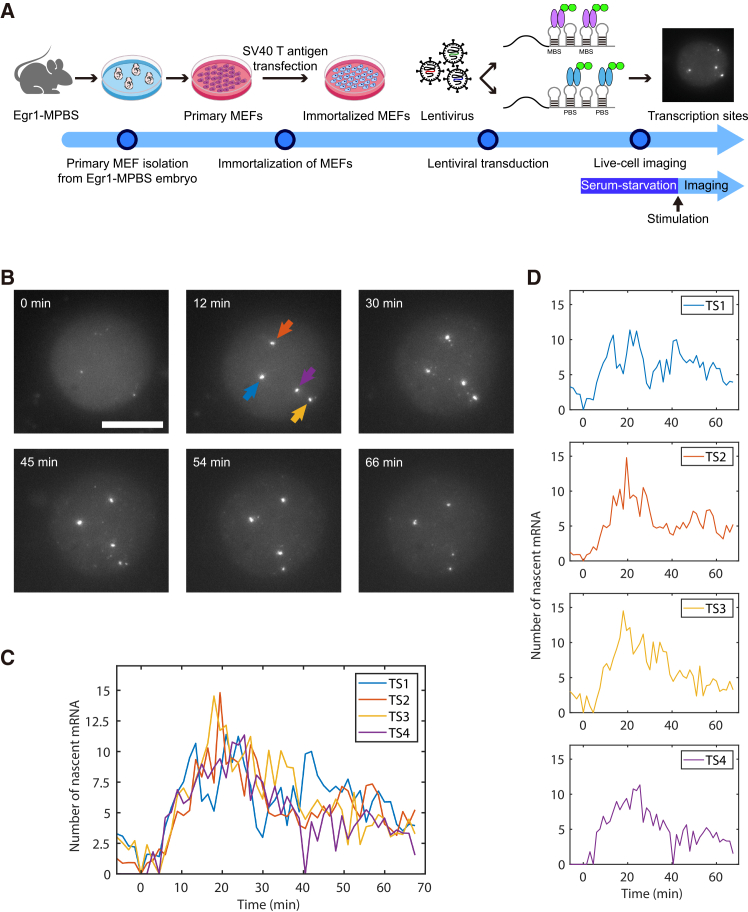
Live-cell imaging of Egr1 transcriptional activity in MEFs (A) Schematic of the experimental setup for real-time imaging of Egr1 transcription in MEFs. MEFs were derived from Egr1-MPBS mice, immortalized with SV40 T antigen, and transduced with lentiviral vectors expressing PCP-GFP or MCP-GFP. Live-cell imaging was performed following serum stimulation. (B) Representative images of Egr1 transcription sites in a nucleus at different time points (Video S1). Arrows indicate the position of active transcription sites. Note that the contrast was optimized for visualizing nuclear transcription sites. Thus, individual cytoplasmic mRNAs are not readily visible due to the wide dynamic range. Scale bars, 10 μm. (C and D) Quantification of fluorescence intensity profiles for individual transcription sites indicated in (B).

To investigate the transcriptional dynamics of the endogenous Egr1 gene in real time, we performed live-cell imaging in Egr1-MPBS MEFs. Cells were serum-starved overnight and then stimulated with 15% FBS to induce Egr1 expression. Imaging was conducted before and after stimulation in Egr1-MPBS × MCP and Egr1-MPBS × PCP MEFs (Figure 4A). In both cell lines, up to four active transcription sites were detected per nucleus (Figure 4B and Video S1), consistent with the smFISH results in Figure 2B. We tracked the position and fluorescence intensity of each transcription site over time to quantify transcriptional activity in real-time.

Video S1. Transcriptional activity of Egr1 upon serum stimulation (corresponding to Figure 4B) The Egr1-MPBS × MCP MEF cell exhibits the emergence of transcription sites within a few minutes after serum stimulation. Up to four transcription sites are detected, each producing individual mRNAs. Images were acquired every 90 s and are displayed at 360× real time. Scale bars, 10 μm.

In some cells, both single mRNAs and active transcription sites were observed simultaneously in the nucleus. These cells allowed us to estimate the number of nascent transcripts at each transcription site by normalizing transcription site intensities to the average intensity of single mRNAs (Figure 4C). In this representative cell, all four alleles were activated by serum stimulation but displayed distinct temporal profiles of transcriptional activity, reflecting allele-specific dynamics (Figure 4D). These data demonstrate the feasibility of directly counting the number of nascent transcripts at individual transcription sites using single nuclear mRNAs as a reference. Overall, our live-cell imaging approach provides direct quantitative insight into allele-specific Egr1 transcriptional dynamics at single-molecule resolution.

### Quantitative analysis of Egr1 transcriptional bursting

**Figure 5:**
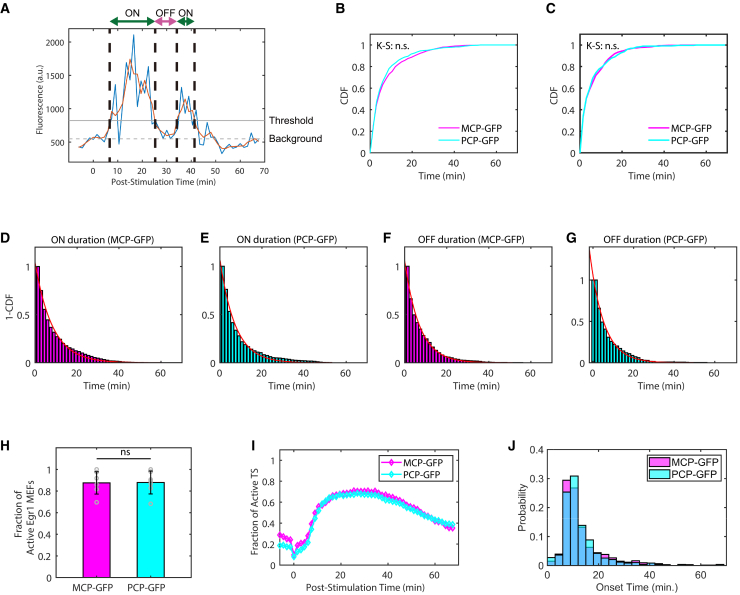
Comparison of MCP-GFP and PCP-GFP labeling for live-cell imaging of Egr1 transcription (A) Representative fluorescence intensity trace of a single Egr1 transcription site (Video S2). The blue line shows the raw signal (captured at 90 s intervals), the orange line a rolling average over three frames, the dashed gray line the nuclear background, and the solid gray line the ON/OFF threshold (1.5× background). (B and C) Cumulative distribution functions (CDFs) of transcriptional ON (B) and OFF (C) durations in Egr1-MPBS × PCP and Egr1-MPBS × MCP MEFs. Distributions were compared using the Kolmogorov-Smirnov (K-S) test, with no significant differences detected (*p* > 0.05). (D–G) Inverse cumulative histograms of ON and OFF durations (*n* = 834 ON and *n* = 479 OFF events from 240 nuclei, Egr1-MPBS × PCP; *n* = 1135 ON, *n* = 596 OFF events from 298 nuclei, Egr1-MPBS × MCP). Each distribution was fitted with a single-exponential decay model. (H) Fraction of MEFs exhibiting at least one active transcription site. The mean fraction was 0.88 ± 0.04 (SEM) for both MCP- and PCP-labeled cells (*t* test, *p* > 0.05; *n* = 240 nuclei, PCP-GFP; *n* = 298 nuclei, MCP-GFP). (I) Time course of the average proportion of active transcription sites per cell (normalized to a maximum of four). No significant difference was observed between groups. (J) Probability histograms of transcription onset times for MCP- (*n* = 605) and PCP-labeled sites (*n* = 576), showing comparable activation timing. Error bars represent SEM.

Analysis of live-cell imaging traces showed that individual Egr1 transcription sites undergo cycles of activation (ON) and inactivation (OFF) following serum stimulation (Figure 5A). This switching behavior is characteristic of transcriptional bursting, which arises from dynamic transitions between active and inactive transcriptional states, a widely observed phenomenon from bacteria to mammals.^38^^,^^39^ Transcriptional bursting of Egr1 was quantified by thresholding fluorescence intensity, defining ON states as > 1.5-fold above background and OFF states otherwise. The durations of ON and OFF states were measured across all tracked transcription sites to characterize the temporal dynamics of Egr1 transcription after serum stimulation.

Video S2. Transcription bursting of Egr1 upon serum stimulation (corresponding to Figure 5A) An Egr1-MPBS × MCP MEF cell exhibits transcription bursting activity induced by serum stimulation. The transcription site activity is characterized by ON and OFF durations, determined by a signal intensity threshold calculated from the nucleus background. Images were acquired every 90 s and are displayed at 360× real time. Scale bars, 10 μm.

We compared MEFs expressing MCP-GFP or PCP-GFP to assess whether the labeling strategy affected bursting behavior. The distributions of ON and OFF durations were highly similar between the two groups (Figures S2A and S2B). We analyzed these distributions using the cumulative distribution function (CDF), which revealed no significant differences for either ON or OFF durations (Figures 5B and 5C). Inverse cumulative histograms of ON and OFF durations were well fitted by single-exponential functions (Figures 5D–5G), yielding ON time constants (τ) of 8.2 min for MCP-GFP and 7.3 min for PCP-GFP (Figures 5D and 5E), and OFF time constants of 7.0 min and 6.9 min, respectively (Figures 5F and 5G). Mean ON and OFF durations calculated directly from individual bursts were comparable to these fitted values (Table S2). Collectively, these results indicate that MCP- and PCP-based labeling provide consistent measurements of Egr1 transcriptional bursting kinetics.

To further characterize the transcriptional response at the cellular level, we analyzed the fraction of active cells and transcription sites over time. A cell was classified as active if at least one transcription site was detected during imaging. The average fraction of active cells was 0.88 ± 0.04 for both MCP-GFP and PCP-GFP (mean ± SEM; Figure 5H), indicating comparable transcriptional responsiveness between the two labeling systems. We next quantified the fraction of active transcription sites over time. Both MCP- and PCP-labeled MEFs exhibited similar temporal profiles, with transcription site activity peaking between 15 and 30 min after serum stimulation and gradually declining thereafter (Figure 5I). These results are consistent with our smFISH analyses and indicate that the tagging method does not alter the overall temporal activation of Egr1.

Finally, to assess the onset of transcription, we defined induction time as the time point when a transcription site first appeared after stimulation. Both MCP-GFP and PCP-GFP-expressing cells showed a rapid increase in transcriptional activity, with most sites activating within 15 min (Figure S2C). The distribution of induction times was essentially indistinguishable between labeling strategies (Figure 5J), further confirming that MCP- and PCP-based labeling report Egr1 transcriptional activation with comparable fidelity.

### Quantitative comparison of endogenous Egr1 mRNA diffusion dynamics in the nucleus and cytoplasm

**Figure 6:**
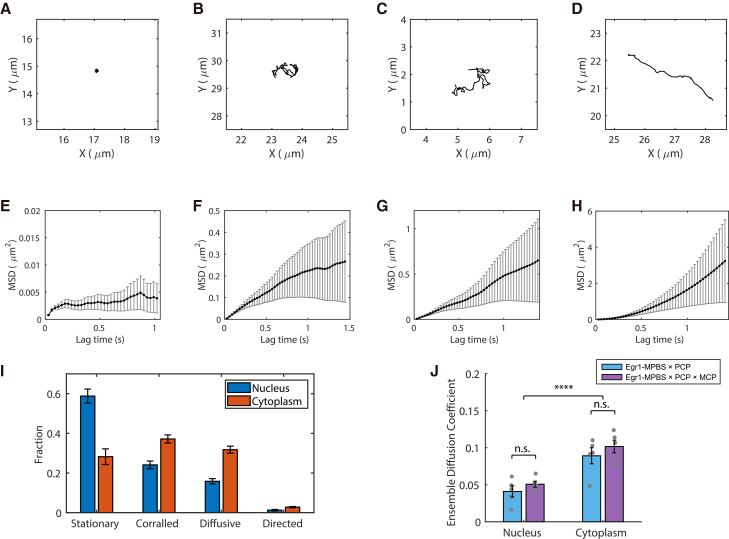
Quantitative analysis of Egr1 mRNA diffusion in the nucleus and cytoplasm Egr1 mRNA trajectories were classified into four motion types: stationary, corralled, diffusive, and directed. (A–D) Representative trajectories for each motion type. (E–H) Mean squared displacement (MSD) versus lag-time plots for the trajectories shown in (A–D). (I) Fraction of Egr1 mRNAs exhibiting each motion type. Representative live-cell imaging videos are shown for the nucleus (Video S3) and cytoplasm (Video S4). (J) Average ensemble diffusion coefficients of Egr1 mRNAs labeled with PCP-GFP or PCP-GFP × MCP-GFP in the nucleus and cytoplasm (mean ± SEM; *p* > 0.05). Diffusion coefficients were significantly higher in the cytoplasm compared with the nucleus (∗∗∗∗*p* < 0.0001). Error bars represent SEM.

We next investigated the intracellular mobility of endogenous Egr1 mRNAs by tracking single molecules in the nucleus (Video S3) and cytoplasm (Video S4) of MEFs. Individual trajectories were classified into four motion states: stationary, corralled, diffusive, or directed (Figures 6A–6H) using previously established methods.^21^^,^^40^^,^^41^ This analysis revealed clear differences between nuclear and cytoplasmic populations (Figure 6I). In Egr1-MPBS × PCP MEFs, nuclear mRNAs exhibited a higher fraction of stationary motion (∼58%) than cytoplasmic mRNAs (∼30%). Conversely, corralled and diffusive motion were more prevalent in the cytoplasm (37% and 31%, respectively) than in the nucleus (25% and 16%). Directed motion was rare overall but occurred slightly more often in the cytoplasm (∼2.7%) than in the nucleus (∼1.3%).

Video S3. Diffusion of Egr1 mRNAs in the nucleus (corresponding to Figure 6I) After transcription, newly synthesized mRNAs diffuse within the nucleus. Images were acquired every 30 ms and are displayed at 3× real time. Scale bars, 10 μm.

Video S4. Diffusion of Egr1 mRNAs in the cytoplasm (corresponding to Figure 6I) After nuclear export, mRNA molecules diffuse throughout the cytoplasm. Images were acquired every 30 ms and are displayed at 3× real time. Scale bars, 10 μm.

We then measured ensemble diffusion coefficients in MEFs where Egr1 mRNAs were labeled with PCP-GFP alone (Egr1-MPBS × PCP) or with both PCP-GFP and MCP-GFP (Egr1-MPBS × PCP × MCP) (Figure 6J). Trajectories with ≥14 time points were analyzed, and diffusion coefficients (D) were calculated by linear fitting of mean squared displacement (MSD) at time lags of 2Δt, 3Δt, and 4Δt. In the nucleus, average ensemble diffusion coefficients were 0.041 ± 0.008 μm^2^/s for Egr1-MPBS × PCP (*n* = 33 cells) and 0.051 ± 0.004 μm^2^/s for Egr1-MPBS × PCP × MCP (*n* = 40 cells) (mean ± SEM), which were comparable to previous measurements in other systems.^41^^,^^42^^,^^43^ In the cytoplasm, diffusion was significantly faster, with coefficients of 0.089 ± 0.011 μm^2^/s for Egr1-MPBS × PCP (*n* = 67 cells) and 0.102 ± 0.009 μm^2^/s for Egr1-MPBS × PCP × MCP (*n* = 66 cells) in line with earlier studies.^21^^,^^40^ Overall, ensemble diffusion coefficients were significantly higher in the cytoplasm compared with the nucleus (*t* test, ∗∗∗∗*p* < 0.0001). No statistically significant differences were observed between the two labeling conditions in either compartment (*t* test, *p* > 0.05). In cells expressing PCP-GFP alone, each Egr1 transcript could be tagged with up to 24 GFP molecules, whereas in cells co-expressing PCP-GFP and MCP-GFP, as many as 48 GFPs could be recruited to a single mRNA. This corresponds to roughly a 2-fold increase in tag mass per transcript, yet diffusion coefficients remained unchanged. This likely reflects the fact that endogenous Egr1 mRNAs move as large messenger ribonucleoprotein (mRNP) complexes already bound by numerous RNA-binding proteins and ribosomes, such that the additional GFP load contributes negligibly to overall mobility.

To further examine mRNA dynamics, we analyzed the distributions of diffusion coefficients across individual trajectories (Figure S3). The distributions were highly similar between Egr1-MPBS × PCP and Egr1-MPBS × PCP × MCP MEF mRNAs within each compartment, further confirming that increasing the number of GFP tags does not measurably alter diffusion properties. The cytoplasm exhibited broader distributions with a long tail toward higher values, indicating more heterogeneous mobility, whereas the nuclear distributions were narrower and shifted toward lower values. Taken together, these findings highlight distinct compartment-specific diffusion behaviors of endogenous Egr1 mRNAs, with nuclear motion dominated by stationary and corralled states and cytoplasmic motion characterized by higher heterogeneity and increased diffusivity.

## Discussion

Understanding how IEGs such as Egr1 are dynamically regulated requires tools that can capture transcriptional and post-transcriptional events in real time without perturbing endogenous control. In this study, we generated and validated a novel knock-in mouse model, Egr1-MPBS, which enables live-cell imaging of Egr1 transcription and mRNA dynamics. By integrating MS2 and PP7 stem-loop cassettes into the 3′UTR of Egr1 and expressing the corresponding MCP-GFP and PCP-GFP proteins, this model allows real-time visualization of transcriptional activity and tracking of individual transcripts in both the nucleus and cytoplasm. This system preserves endogenous gene regulation while providing single-molecule resolution, offering a robust platform for dissecting IEG dynamics.

An important design consideration of the Egr1-MPBS mouse was the use of an alternating 12× MS2/PP7 binding site configuration rather than a single high-copy array. This dual architecture enables orthogonal recruitment of distinct RNA-binding proteins, a strategy previously shown to improve signal robustness and reduce background in RNA imaging applications.^34^^,^^35^ Moreover, dual MS2/PP7 systems provide a modular platform that allows simultaneous transcript visualization and recruitment of functional protein assemblies.^36^ Importantly, our data demonstrate that the 12× MPBS configuration provides sufficient signal-to-noise ratio for the reliable detection of endogenous Egr1 transcripts without perturbing transcriptional kinetics or mRNA stability. Thus, this design was selected not merely for signal amplification but to enable combinatorial and orthogonal protein recruitment strategies compatible with future *in vivo* applications.

Validation experiments confirmed correct integration of 12× MPBS cassettes and showed that the cassette insertion and labeling strategies did not perturb the transcriptional response of Egr1 to serum stimulation. Live-cell imaging further revealed that Egr1 transcription occurs in stochastic bursts with alternating ON and OFF states. Quantitative analysis of bursting kinetics showed that Egr1 exhibits temporal patterns distinct from those reported for other genes such as Arc and β-actin,^23^^,^^28^^,^^32^ highlighting diversity in bursting behaviors across genes. These results establish Egr1 as a useful model for exploring the mechanisms underlying transcriptional variability among IEGs. While Arc is primarily associated with synaptic plasticity, Egr1 acts as a broader master regulator of cell growth and differentiation. Therefore, the Egr1-MPBS model provides a unique opportunity to study how different signaling pathways converge to tune transcriptional frequency and amplitude at the single-allele level in an endogenous context.

An important aspect of this work was to benchmark MS2- and PP7-based labeling systems under identical genomic conditions. Across all measured parameters, including ON/OFF durations, transcriptional induction timing, and the fraction of active alleles, MCP-GFP and PCP-GFP yielded indistinguishable results. This demonstrates that the two systems are functionally interchangeable and validates their use for live-cell RNA imaging. Importantly, this comparability enhances the reliability of transcriptional measurements and provides cross-validation across different studies employing either labeling strategy.

Beyond transcriptional regulation, our model allowed quantitative analysis of mRNA mobility. By tracking single transcripts, we classified nuclear and cytoplasmic trajectories into four motion types and found marked differences between compartments. Nuclear transcripts were largely stationary or corralled, whereas cytoplasmic transcripts exhibited more heterogeneous and faster diffusion. Increasing the GFP load from 24 (PCP only) to 48 (PCP and MCP) tags per transcript did not measurably affect diffusion, consistent with the notion that mRNAs move as large mRNP complexes, where the relative contribution of GFP mass is negligible.^22^^,^^44^ These observations show that our model enables simultaneous interrogation of transcriptional activity and post-transcriptional RNA trafficking in intact cells.

As an IEG and transcription factor, Egr1 plays critical roles in activity-dependent processes such as synaptic plasticity, learning, and memory. The ability to monitor Egr1 transcription in real time and to quantify its mRNA mobility across nuclear and cytoplasmic compartments opens new avenues for studying the spatiotemporal regulation of gene expression in the context of neural activity and behavior. Our study thus extends the scope of live-cell RNA imaging and provides a versatile platform for investigating IEG regulation in physiologically relevant contexts. Importantly, this work builds on our previous efforts to visualize endogenous Arc mRNA dynamics in the intact mouse brain during virtual reality navigation,^29^ where activity-dependent transcriptional regulation was revealed in behaving animals. By establishing the Egr1-MPBS model, we now add a complementary tool for examining another key IEG with distinct regulatory kinetics, enabling cross-gene comparisons of transcriptional bursting and mRNA trafficking *in vivo*. By rigorously establishing this system in mammalian cells first, we provide the essential foundation for moving toward circuit-level investigations in intact tissues—a level of complexity where the differences between endogenous regulation and exogenous reporters become most critical. Together, these advances highlight the potential of genetically encoded RNA labeling systems to connect single-molecule transcriptional dynamics with circuit-level function and behavior.

While the MS2 and PP7 systems remain powerful tools for imaging single mRNAs, a well-recognized limitation is the background fluorescence generated by unbound coat protein fusions. Several strategies have been developed to address this issue, including split-protein complementation systems in which RNA-binding proteins and fluorescent proteins are divided into fragments that reconstitute a signal only upon simultaneous binding to the target RNA. This principle has been demonstrated in bacteria^45^^,^^46^ and mammalian cells.^34^^,^^35^^,^^47^^,^^48^ In particular, the use of superfolder GFP fragments fused to MCP and PCP has enabled background-free detection of MPBS-tagged transcripts in both the nucleus and the cytoplasm.^35^ In addition to fluorescence-based approaches, split-luciferase systems have emerged as promising alternatives. Recent work demonstrated the feasibility of using split NanoLuc fused to MCP and PCP (“RNA lanterns”) for bioluminescent RNA tracking in cells and live animals.^49^ These advances highlight the potential for developing next-generation, background-free RNA imaging systems, with the Egr1-MPBS model serving as a powerful testbed for *in vivo* validation.

In conclusion, the Egr1-MPBS mouse model provides a robust system for visualizing transcriptional bursting and quantifying mRNA mobility of an IEG at single-molecule resolution. By validating the equivalence of MS2- and PP7-based labeling and linking this framework to emerging background-free imaging strategies, our work paves the way toward reliable single-RNA visualization *in vivo*. Ultimately, this platform will facilitate mechanistic studies of transcriptional regulation and RNA trafficking in the intact brain, enabling direct connections between molecular dynamics, neural activity, and behavior.

### Limitations of the study

Although the Egr1-MPBS mouse model provides a robust platform for real-time monitoring of endogenous mRNA, several limitations remain to be addressed. The primary limitation is that our current characterization of the system was conducted in immortalized MEFs, rather than in intact tissues or living animals. While these cell-line experiments allowed for the precise quantification of Egr1 transcriptional bursting and mRNA diffusion kinetics, they do not fully capture the physiological complexity of Egr1 regulation in its native context, such as the brain during learning and memory tasks. Given that this mouse model was specifically designed for *in vivo* applications, further studies are required to validate its performance in specialized cell types such as neurons within their anatomical niche. Additionally, the current imaging setup relies on the constitutive expression of MCP- or PCP-GFP, which can result in background fluorescence. Integrating more advanced techniques, such as split-GFP systems to minimize background noise, will be essential to fully leverage the potential of this model for high-resolution, background-free imaging *in vivo*.

## Resource availability

### Lead contact

Requests for further information and resources should be directed to and will be fulfilled by the lead contact, Hye Yoon Park (hyp@umn.edu).

### Materials availability

All unique reagents generated in this study, including the transgenic mouse line and derived MEF cell lines, are available from the lead contact upon reasonable request.

### Data and code availability

• Data: All data reported in this paper will be shared by the lead contact upon reasonable request.
• Data: This paper does not report original code.
• For other items: Any additional information required to reanalyze the data reported in this paper is available from the lead contact upon request.

## Acknowledgments

This work was supported by the National Research Foundation (NRF) of Korea grant no. 2020R1A2C2007285 and the startup fund from the 10.13039/100007249University of Minnesota. Figure 4A was partly generated from adapted figures provided by Servier Medical Art (https://smart.servier.com/), licensed under CC BY 4.0 (https://creativecommons.org/licenses/by/4.0/) and bioicons (https://bioicons.com/), licensed under CC BY 3.0 (https://creativecommons.org/licenses/by/3.0/).

## Author contributions

H.Y.P. conceived and supervised the project. H.A. performed the experiments, developed analysis scripts, and analyzed the data. H.J. assisted with single-molecule FISH experiments. D.W.K. generated immortalized MEF cell lines. J.Y.S. assisted with transgenic mouse generation. H.A. and H.Y.P. wrote the manuscript.

## Declaration of interests

The authors declare that no competing interests exist.

## Declaration of generative AI and AI-assisted technologies in the writing process

During the preparation of this work, the authors used Gemini to improve the readability of some texts. After using this tool, the authors reviewed and edited the content as needed and take full responsibility for the content of the publication.

## STAR★Methods

### Key resources table

**Table:** 

REAGENT or RESOURCE	SOURCE	IDENTIFIER
**Antibodies**		

Anti-GFP antibody (Chicken polyclonal)	Aves Lab	Cat# GFP-1020; RRID: AB_2307313
Alexa Fluor 647-conjugated goat anti-chicken IgY (H + L) secondary antibody (Goat polyclonal)	Invitrogen	Cat# A21449; RRID: AB_2535866

**Chemicals, peptides, and recombinant proteins**		

Dulbecco’s modified Eagle’s medium (DMEM)	Gibco	Cat# 11995065
Fetal bovine serum (FBS)	Gibco	Cat # A5669801
Penicillin/streptomycin (Pen-Strep)	Gibco	Cat # 15140122
GlutaMax	Gibco	Cat # 35050061
Trypsin-EDTA (0.25%)	Gibco	Cat# 2500072
Trypsin-EDTA (0.05%)	Gibco	Cat# 25300054
DPBS	Gibco	Cat# 14190144
Opti-MEM	Gibco	Cat# 31985062
D,L-Sulforaphane (SFN)	Sigma-Aldrich	Cat# S4441
PBS	Fisher Bioreagent	Cat# BP3991
5,6-dichlorobenzimidazole 1-β-D-ribofuranoside (DRB)	Sigma-Aldrich	Cat# 287891-50 MG
Magnesium chloride (MgCl_2_)	Sigma-Aldrich	Cat# M1028
Calcium chloride (CaCl_2_)	Sigma-Aldrich	Cat# 21115
Paraformaldehyde (PFA)	Electron Microscopy Sciences	Cat# 15714
Glycine	Bio-Rad	Cat# 1610717
Triton X-100	Thermo Fisher Scientific	Cat# 28314
Formamide	Sigma-Aldrich	Cat# F9037-100 mL
Saline sodium citrate (SSC) buffer	Invitrogen	Cat# AM9763
Bovine serum albumin (BSA)	Sigma	Cat# A7906
Bovine serum albumin (BSA)	Roche	Cat# 10711454001
Dextran sulfate	Sigma-Aldrich	Cat# D8906
single-stranded DNA (ssDNA)	Sigma-Aldrich	Cat# D7656
tRNA	Sigma-Aldrich	Cat# R1753
Vanadyl ribonucleoside complex	Sigma-Aldrich	Cat# R3380
SUPERase-In	Invitrogen	Cat# AM2694
DAPI	Thermo Fisher Scientific	Cat# 62248
Prolong Gold Antifade Mountant	Invitrogen	Cat# P36965
Leibovitz’s L-15 medium	Gibco	Cat# 21083027

**Critical commercial assays**		

Lipofectamine 2000 reagent	Invitrogen	Cat# 11668019
TriZol™ reagent	Invitrogen	Cat# 15596026
TURBO DNA-free kit	Invitrogen	Cat# AM1907
SuperScript™ IV First-Strand Synthesis System	Invitrogen	Cat # 18091050
TaqMan™ Fast Advanced Master Mix	Applied Biosystems	Cat# 4444556
TaqMan gene expression assay (Egr1)	Applied Biosystems	Assay ID: Mm00656724_m1
TaqMan gene expression assay (18S rRNA)	Applied Biosystems	Assay ID: Mn04277571_s1

**Experimental models: Cell lines**		

Mouse embryonic fibroblasts (MEFs) derived from Egr1-MPBS mice	This paper	N/A

**Experimental models: Organisms/strains**		

Egr1-MPBS mice	This paper	N/A

**Oligonucleotides**		

Primers for Egr1-MPBS forward (5′-GAT CTC AGA GCC AAG TCC TTC -3′)	This paper	N/A
Primers for Egr1-MPBS reverse (5′- CTC CCC CAA AGT GAG GAT TTA ACT CC -3')	This paper	N/A
Egr1 FISH probe	This paper	Sequences provided in Table S1
MPBS FISH probe	This paper	Sequences provided in Table S1
Fos FISH probe	This paper	Sequences provided in Table S1

**Recombinant DNA**		

Ubc-stdMCP-stdGFP	Institute for Basic Science (IBS)	N/A
Ubc-stdPCP-stdGFP	Institute for Basic Science (IBS)	N/A
SV40 large T antigen	This paper	N/A

**Software and algorithms**		

QuantStudio™ Design and Analysis	Applied Biosystems	https://www.thermofisher.com
Micro-manager 2	Open Imaging	https://micro-manager.org/Version_2.0
TrackNTrace	University of Göttingen	https://github.com/scstein/TrackNTrace
MATLAB	MathWorks	https://www.mathworks.com
ImageJ/Fiji	NIH	RRID:SCR_003070
HybTrack	Seoul National University	https://github.com/bhlee1117/HybTrack

### Experimental model and study participant details

Egr1-MPBS knock-in mice were generated on a C57BL/6 background. All animal care and experimental procedures were approved by the Institutional Animal Care and Use Committee (IACUC) of Seoul National University and the University of Minnesota (Protocol ID 2507-43149A). Mouse embryonic fibroblasts (MEFs) were isolated from E14 embryos derived from knock-in mice. For MEF isolation, E14 embryos were harvested without sex selection, and cells from multiple embryos were pooled. Cells were cultured in DMEM supplemented with 10% fetal bovine serum, 1% penicillin-streptomycin, and 1% GlutaMAX at 37°C in a humidified incubator with 5% CO_2_. For immortalization, MEFs were transfected with an SV40 large T antigen expression plasmid. The identity of the immortalized knock-in MEFs was authenticated via PCR-based genotyping to confirm the Egr1-MPBS insertion. Immortalized MEFs were maintained under standard culture conditions (37°C, 5% CO_2_).

### Method details

#### Generation of the Egr1-MPBS mouse

All animal care and experimental procedures were performed according to the protocols approved by the Institutional Animal Care and Use Committee (IACUC) at Seoul National University and the University of Minnesota. The Egr1-MPBS knock-in mouse model was generated using CRISPR/Cas9-mediated genome editing to label the endogenous Egr1 mRNA with 12 repeats of MS2 and PP7 stem-loop pairs inserted into the 3′ untranslated region (3′UTR). The Egr1 gene is located on the forward strand of mouse chromosome 18 and is transcribed into a 3,072-nucleotide (nt) mRNA transcript. Candidate CRISPR guide RNAs (gRNAs) were designed to target the 3′UTR region (ATAAAGAAAA), and the optimal gRNAs were selected based on predicted efficiency and specificity. A double-stranded DNA (dsDNA) donor plasmid containing 1,368-nt MPBS cassette flanked by homology arms was synthesized for homology-directed repair. Microinjection of the CRISPR-Cas9 ribonucleoprotein complex, gRNAs (5′-CTTGATGGTCTAGCGCTGAAGGG-3′, 5′-CTGTACAAAGATGCAGGGCAGGG-3′), and the dsDNA donor into fertilized C57BL/6 mouse zygotes was performed by Macrogen Inc. (Seoul, South Korea). Microinjected embryos were implanted into pseudopregnant females, and potential founder mice were screened by PCR genotyping and confirmed by Sanger sequencing. Heterozygous founders were bred to establish homozygous Egr1-MPBS knock-in mouse lines for subsequent experiments.

#### Genotyping

To genotype the Egr1-MPBS mouse model, PCR was performed using a forward and reverse primer set. The forward primer sequence was 5′-GAT CTC AGA GCC AAG TCC TTC -3′ and the reverse primer sequence was 5'- CTC CCC CAA AGT GAG GAT TTA ACT CC -3′. This PCR yielded a 402 bp product for the WT allele and a 2,100 bp product for the transgenic allele. PCR was conducted at 94°C for 30 s, 65°C for 1 min, and 72°C for 2 min for 35 cycles.

#### Generation of MEF lines from Egr1-MPBS mice

Homozygous Egr1-MPBS mouse embryos were isolated at embryonic day 14 (E14) from Egr1 MPBS homozygous pregnant mice. Embryonic heads were removed using a sterile razor blade, and non-fibroblastic tissues, including internal organs, were carefully dissected out. The remaining body tissues were finely minced and digested in 0.25% trypsin-EDTA (Thermo Fisher Scientific), followed by trituration and incubation at 37°C for 45 min, with gentle pipetting every 15 min to facilitate cell dissociation. Following digestion, trypsin was neutralized with MEF culture medium consisting of Dulbecco’s modified Eagle’s medium (DMEM; Gibco) supplemented with 10% fetal bovine serum (FBS; Gibco), 1% penicillin/streptomycin (Pen-Strep; Gibco), and 1% GlutaMax (Gibco). The cell suspension was centrifuged, and the resulting pellet was resuspended in fresh cell culture medium. Cells were seeded onto 10 cm cell culture dishes and incubated at 37°C in a humidified incubator with 5% CO_2_. When primary MEFs reached 70–90% confluency, MEF primary cells were transfected with an SV40 large T antigen expression plasmid using Lipofectamine 2000 reagent (Invitrogen, 11668019) diluted in serum-free medium (Opti-MEM). After 24 h, the medium was replaced with fresh culture medium, and cells were allowed to proliferate for an additional 48 h. To select for immortalized cells, cultures were treated with 30 μM D,L-Sulforaphane (SFN; Sigma-Aldrich), as SV40-immortalized MEFs exhibit increased resistance to SFN-induced apoptosis compared to wild-type MEFs.^50^ Surviving colonies were expanded and maintained in MEF culture medium at 37°C in a 5% CO_2_ humidified incubator.

#### Lentiviral transduction

Immortalized Egr1-MPBS MEF cells were infected with Ubc-stdMCP-stdGFP or Ubc-stdPCP-stdGFP lentivirus to enable imaging of transcription sites and single mRNA molecules. Following lentiviral infection, cells were harvested in 1× PBS and analyzed for GFP expression by flow cytometry. Fluorescence data were acquired using a FACS Aria II flow cytometer (BD Biosciences), and viable GFP-positive cells were collected for subsequent cell culture and imaging. Lentiviral vectors were produced and provided by the Institute for Basic Science (IBS).

#### qRT-PCR

Immortalized MEFs derived from Egr1-MPBS, Egr1-MPBS × MCP, Egr1-MPBS × PCP, and WT mice were cultured in a 6-well cell culture plate. MEFs were incubated in DMEM (Gibco) supplemented with 10% Fetal Bovine Serum (FBS) (Gibco, A5669801), 1% penicillin-streptomycin (Pen-Strep) (Gibco, 15140122), and 1% GlutaMAX (Gibco, 35050061) until they reached confluency. After overnight starvation in DMEM containing 1% Pen-Strep and 1% GlutaMAX, MEFs were stimulated with 15% FBS for 15 min, washed, and then treated with 0.45 μM 5,6-dichlorobenzimidazole 1-β-D-ribofuranoside (DRB) for 15, 30, 60, 90, 120, or 150 min. Total RNA was isolated from MEFs at different DRB treatment time points using TriZol reagent (Invitrogen, 15596026). To remove contaminating DNA, the TURBO DNA-free kit (Invitrogen, AM1907) was used. cDNA synthesis was performed from DNase-treated total RNA using SuperScript IV First-Strand Synthesis System (Invitrogen, 18091050). The resulting cDNA was then used for qRT-PCR with the TaqMan Fast Advanced Master Mix (Applied Biosystems, 4444556). The TaqMan assays used for the two-step qRT-PCR were Mm00656724_m1 (Egr1) and Mn04277571_s1 (18S rRNA), with 18S rRNA serving as the internal control. All qRT-PCR experiments were conducted using a QuantStudio 5 Real-time PCR system (ThermoFisher). Samples were run in triplicate and analyzed using the QuantStudio Design and Analysis software. Ct values for target and control genes were determined using a threshold set from the amplification phase. Relative expression levels were calculated by first obtaining ΔCt (target-control) and then applying the ΔΔCt method for comparison across samples.

#### Single-molecule fluorescence *in situ* hybridization (smFISH)

smFISH experiments were performed using three immortalized MEF lines: WT, Egr1-MPBS, and Egr1-MPBS × PCP. For stimulated conditions, MEFs were serum-starved overnight and subsequently stimulated with 15% FBS for 0–150 min (0, 15, 30, 60, 90, 120, and 150 min). Following treatment, cells were washed with PBS containing 5 mM MgCl_2_ (PBSM) and fixed with 4% paraformaldehyde (PFA; Electron Microscopy Sciences, 15714) for 20 min. After fixation, cells were washed twice with PBSM and rehydrated by shaking in PBSM for 10 min. Permeabilization was performed by sequential shaking in PBS containing 0.1% Triton X-100 (Thermo Scientific) (PBST) and PBSM, repeated twice. Cells were pre-incubated with 10% formamide in 2× saline sodium citrate (SSC) buffer (Invitrogen, AM9763) for 10 min at room temperature. Hybridization was carried out using a buffer containing 10% bovine serum albumin (BSA; Roche, 10711454001), 2× SSC and 10% dextran sulfate (Sigma-Aldrich, D8906). Two FISH probes were used: one targeting the coding sequence (CDS probes) of Egr1 and another targeting the MS2-PP7 linker region (MPBS probes) (Table S1). Hybridization was performed at 37°C for 3 h or overnight in the presence of single-stranded DNA (ssDNA) (Sigma-Aldrich, D7656) and tRNA (Sigma-Aldrich, R1753) as competitors. Following hybridization, cells were washed twice with warm 10% formamide in 2× SSC and incubated at 37°C for 20 min each. This was followed by a 10-min shaking in 2× SSC at room temperature and another 10-min shaking in PBSM at room temperature. Nuclei were stained with 0.5 μ g/ml DAPI (Thermo Scientific, 62248) for 1 min, washed in PBSM for 10 min, and mounted using Prolong Gold Antifade Mountant (Invitrogen, P36965).

#### Immunofluorescence (IF) - Single-molecule fluorescence *in situ* hybridization (smFISH)

IF-smFISH experiments were performed using immortalized MEF lines (Egr1-MPBS × PCP × MCP). To detect single molecules, MEFs were serum-starved overnight and subsequently stimulated with 15% FBS for 60 min. Following stimulation, cells were washed with PBS containing 1 mM MgCl_2_ and 1 mM CaCl_2_ (PBS-MC) and fixed with ice-cold 4% paraformaldehyde (PFA; Electron Microscopy Sciences, 15714) for 20 min. After fixation, cells were washed twice with PBS-MC and quenched with 50 mM glycine for 15 min. Permeabilization was performed using PBS-MC containing 0.1% Triton X-100 (Thermo Scientific) (PBST), followed by three washes with PBS-MC. Cells were blocked with 0.5% bovine serum albumin (BSA; Sigma, A7906) in PBS-MC for 15 min and pre-incubated with 10% formamide in 2× saline sodium citrate buffer (SSC; Invitrogen, AM9763) supplemented with 0.5% BSA for 30 min at room temperature. Hybridization was performed overnight at 37°C in a hybridization buffer containing: primary antibody against GFP (Aves Lab, GFP-1020), FISH probes targeting the coding sequence (CDS) of Egr1 (or Fos as a control) (Table S1), 10% formamide, 2× SSC, 10% dextran sulfate (Sigma-Aldrich, D8906), 1 mg/mL tRNA (Sigma-Aldrich, R1753), 0.2 mg/mL BSA (Roche, 10711454001), 2 mM vanadyl ribonucleoside complex (Sigma, R3380), 10 U/mL SUPERase-In (Invitrogen, AM2694) in RNase-free water. Post-hybridization, cells were washed twice with warm 10% formamide and 3% BSA in 2× SSC. Cells were then incubated twice with Alexa Fluor 647-conjugated goat anti-chicken secondary antibody (Invitrogen, A21449) in 10% formamide and 2× SSC at 37°C for 20 min each. After four washes in 2× SSC at room temperature, nuclei were stained with 0.5 μ g/ml DAPI (Thermo Scientific, 62248) for 1 min. Finally, cells were washed in PBS-MC for 10 min and mounted using Prolong Gold Antifade Mountant (Invitrogen, P36965).

#### Live-cell imaging of transcription

Immortalized Egr1-MPBS × MCP and Egr1-MPBS × PCP MEF lines were cultured on confocal imaging dishes. Cells were incubated in DMEM supplemented with 10% FBS, 1% Pen-Strep, and 1% GlutaMAX until reaching 60–70% confluency. For stimulation experiments, cells were serum-starved overnight in DMEM containing 1% Pen-Strep and 1% GlutaMAX. Prior to imaging, the culture medium was replaced with L-15 medium (Gibco, 21083027) supplemented with 1% Pen-Strep and 1% GlutaMAX, Cells were maintained at 37°C during imaging using a temperature-controlled chamber (LCI, Incubator System T), and multiple fields of view containing Egr1-expressing nuclei were selected using a wide-field fluorescence microscope. Cells were stimulated with 15% FBS, and time-lapse imaging was initiated immediately following serum addition. Time-lapse z-stacks were acquired at 90-s intervals with an exposure time of 150 ms per frame and a z-step size of 0.5 μm, for a total of 50 frames per position.

All fluorescence images were acquired using an Olympus IX-83 inverted microscope equipped with a 150×/1.45 NA oil immersion objective (Olympus, UAPON150XOTIRF), an sCMOS EMCCD camera (Teledyne, KINETIX), a SOLA Light Engine III (Lumencor), and an XY motorized stage (Märzhäuser Wetzlar, Tango 2 desktop). An eGFP filter set was used for fluorescence detection. Image acquisition was controlled using Micro-manager 2 software.

#### Live-cell imaging of single mRNA diffusion

To generate a double-labeled cell line (Egr1-MPBS × PCP × MCP), Egr1-MPBS × PCP MEFs were transduced with a Ubc-stdMCP-stdGFP lentivirus. MEFs were cultured on confocal imaging dishes in DMEM supplemented with 10% FBS, 1% Pen-Strep, and 1% GlutaMAX until reaching 60–70% confluency. For stimulation experiments, cells were serum-starved overnight in DMEM containing 1% Pen-Strep and 1% GlutaMAX. Prior to imaging, the culture medium was replaced with L-15 medium (Gibco, 21083027) supplemented with 1% Pen-Strep and 1% GlutaMAX, Cells were maintained at 37°C during imaging using a temperature-controlled chamber (LCI, Incubator System T). To stimulate cells, 15% FBS was added at least 30 min before imaging. Single-molecule dynamics in the nucleus and cytoplasm were monitored by streaming acquisition, with 2000 consecutive frames collected per position at 30-ms exposure, using the same microscope setup described above.

#### Image analysis for smFISH

To analyze smFISH images, multi-channel z stack images were examined to delineate nuclear and cytoplasmic regions for each cell. Nuclear boundaries were identified using DAPI staining, and regions of interest (ROIs) for the nucleus and cytoplasm were manually defined using Fiji software.^51^ The genomic distance of Egr1 CDS and MPBS probes range from 200 to 4,000 bp. The number and spatial distribution of Egr1 transcription sites and individual mRNA molecules were quantified using TrackNTrace software.^52^ Spot detection was performed on z stack images (acquired at 0.25 μ m intervals) using a normalized cross-correlation method, followed by refinement using GPU-accelerated Gaussian maximum likelihood estimation (GPU-Gauss MLE). Detected spots were defined as co-localized if they were within the diffraction limit (300 nm). Candidate signals were classified as valid if they appeared at nearly identical positions across at least three consecutive z slices. Validated signals were further classified as transcription sites or individual mRNA molecules based on intensity and subcellular localization. All quantitative analyses of RNA signals were performed using custom MATLAB scripts.

#### Image analysis for transcription site dynamics from live-cell imaging

To analyze live-cell imaging data, z-stacks were cropped to isolate individual cells. Image processing was performed using Fiji,^51^ including Z-projection, bleach correction, and background subtraction. Up to four transcription sites per cell were detected using TrackNTrace,^52^ and their positions were labeled to determine the number of active sites in each time frame. Fluorescence intensity traces and corresponding background levels were then extracted from the labeled positions using HybTrack.^25^ Transcription activity was classified as “ON” or “OFF” based on an intensity threshold. A site was considered “ON” when its fluorescence intensity exceeded 1.5-fold above the local background for at least 1.5 min.

The transcriptional ON and OFF times were quantified for each cell and represented as cumulative distribution functions (CDFs). Statistical comparisons between Egr1-MPBS × PCP and Egr1-MPBS × MCP cells were performed using the Kolmogorov-Smirnov (K-S) test. ON and OFF time distributions were fitted to single-exponential decay models, and characteristic durations were derived from the fitted exponential coefficients.

The fraction of MEFs exhibiting at least one active transcription site was calculated for both Egr1-MPBS × PCP and Egr1-MPBS × MCP cells. The average proportion of active transcription sites per cell (up to a maximum of four) was quantified and plotted over time. For each transcription site, the onset time, which is defined as the first transition into the ON state, was determined, and the distribution of onset times was summarized as a probability histogram. All averages were calculated on a per-dish basis.

#### Image analysis for single-mRNA diffusion

Single mRNA particles were detected using TrackNTrace.^52^ Particle trajectories were linked if the displacement between consecutive frames was <25 pixels and the gaps due to missed detections were <3 frames. Gaps shorter than 3 frames were interpolated. Only trajectories containing ≥14 consecutive frames were included in the analysis. For each trajectory, the MSD was calculated, and diffusion coefficients (D) were estimated by linear fitting of MSD values at 2Δt, 3Δt, and 4Δt, weighted by their respective errors. Motion states were classified using custom MATLAB scripts: trajectories with MSD <0.025 μ m^2^ were defined as stationary; trajectories in which the slope of the next three time-lag points in the MSD was less than D were classified as corralled; trajectories with unidirectional displacements >1.5 μm were defined as directed; and all remaining trajectories were considered diffusive.

### Quantification and statistical analysis

Statistical details for every comparison, including the specific test performed, sample size (n), the nature of n, measures of central tendency (e.g., mean or median), and the error bar definitions, are provided within the corresponding figure legends. Statistical analyses and data processing were performed using MATLAB (MathWorks), including the HybTrack and TrackNTrace software, and ImageJ (NIH). Data are presented as mean ± SD or mean ± SEM, as indicated in the figure legends. For comparisons involving multiple groups, a two-way analysis of variance (ANOVA) (Figure 1C) or a one-way ANOVA (Figures 3B–3D) was performed. Correlations between two variables were assessed using Pearson correlation coefficients (r) (Figures 3E–3H). Comparisons of cumulative distribution functions (CDFs) were performed using the Kolmogorov-Smirnov (K-S) test (Figures 5B and 5C). Student’s t-tests were used for paired or unpaired comparisons as appropriate (Figures 5H, 6J, and S2C). Statistical significance was defined as *p* < 0.05. Significance levels are indicated as follows: ∗*p* < 0.05, ∗∗*p* < 0.01, ∗∗∗*p* < 0.001, and ∗∗∗∗*p* < 0.0001); n.s. indicates not significant.
