# Supplementary material for: Live-cell imaging of endogenous Egr1 mRNA using an MPBS knock-in mouse model
Source: iScience. 2026 Apr 30;29(6):115955. doi: 10.1016/j.isci.2026.115955 (PMC13197786; doi:10.1016/j.isci.2026.115955)
Supplement: Document S1. Figures S1–S3, Tables S1 and S2 [file mmc1.pdf]

iScience, Volume *29*

Supplemental information

Live-cell imaging of endogenous Egr1 mRNA using an MPBS knock-in mouse model

Hyerim Ahn, Hyeonjeong Jeong, Dong Wook Kim, Jae Youn Shim, and Hye Yoon Park

Table S1. Sequence of the probes used for single-molecule fluorescence in situ hybridization (smFISH), Related to Figure 2.

Probe sequence name	Sequence (5' to 3')	Reporter Dye
Egr1 Probe_1	AGAACTGATGTTGGGTGGTG	Q570
Egr1 Probe_2	AGCGGAGACATCAATTGCAT	Q570
Egr1 Probe_3	GAGTGAGGAAAGGAGCCGAA	Q570
Egr1 Probe_4	AAAGGACTCTGTGGTCAGGT	Q570
Egr1 Probe_5	ATCGCCTTCTCATTATTCAG	Q570
Egr1 Probe_6	AGTCGTTTGGCTGGGATAAC	Q570
Egr1 Probe_7	TATAGGTGATGGGAGGCAAC	Q570
Egr1 Probe_8	ACAAAGTGTTGCCACTGTTG	Q570
Egr1 Probe_9	CTAGGCTGAAAAGGGGTTCA	Q570
Egr1 Probe_10	TGAAGAGGTCGGAGGATTGG	Q570
Egr1 Probe_11	GAGTAGATGGGACTGCTGTC	Q570
Egr1 Probe_12	GTTGGGAGTAGGAAAGGTGG	Q570
Egr1 Probe_13	GGGGCTCAGGAAAAATGTCA	Q570
Egr1 Probe_14	ATCATGGGAACCTGGAAACC	Q570
Egr1 Probe_15	CCTGTTGTTGTGGAAACAGA	Q570
Egr1 Probe_16	TGGATAGTGGAGTGAGCGAA	Q570
Egr1 Probe_17	TGAGTGGCGAAGGCTTTAAT	Q570
Egr1 Probe_18	TGATGAGCTGGGATTGGTAG	Q570
Egr1 Probe_19	CAAGCATATGGGCGTTCATG	Q570
Egr1 Probe_20	GAGAAAAGCGGCGATCGCAG	Q570
Egr1 Probe_21	ATATGGCGGGTAAGCTCATC	Q570
Egr1 Probe_22	TTACGCATGCAGATTCGACA	Q570
Egr1 Probe_23	AAGGTGGTCACTACGACTGA	Q570
Egr1 Probe_24	AAACTTCCTCCCACAAATGT	Q570
Egr1 Probe_25	AATGGATTTTGGTATGCCTC	Q570
Egr1 Probe_26	CACTTTTGTCTGCTTTCTTG	Q570
Egr1 Probe_27	GAAAGCAGGTGGAACGGAGG	Q570
Egr1 Probe_28	GTCGCTGTCATGTCTGAAAG	Q570
Egr1 Probe_29	ATTTCAATTGTCCTGGGAGA	Q570
Egr1 Probe_30	TGTGCTTTTATGTCTTTCCT	Q570
Egr1 Probe_31	CCTTCTACTCGTGAGTAGAA	Q570
Egr1 Probe_32	GCAGCTGAAGTCAAAGGGAA	Q570
Egr1 Probe_33	GTCCTTTGGATAGAGGTGAA	Q570
Egr1 Probe_34	GATTTATCCAATACCATGCA	Q570
Egr1 Probe_35	ATGGCACAGATGCTGTACAA	Q570

Egr1 Probe_36	GAGGATCATCATTGGTTTGC	Q570
Egr1 Probe_37	TCACAGCAGAGTCATCACAA	Q570
Egr1 Probe_38	TGTGAGAGTTACAGTCGAGC	Q570
Egr1 Probe_39	TTGAAACCAAAGGGACGGGC	Q570
Egr1 Probe_40	GCAAGGCGTGTCACACAAAA	Q570
Egr1 Probe_41	TCACATCTGCGCATGTCAAG	Q570
Egr1 Probe_42	TTAAGGCTAAGGTGAGCGTG	Q570
Egr1 Probe_43	ATTCTGGAGACCGAAAGCTC	Q570
Egr1 Probe_44	TCCAACTCCTGAATAGATGT	Q570
Egr1 Probe_45	CTACTCAGTAGGTAACCACA	Q570
Egr1 Probe_46	GCCAAACAGGTTACTTTGTT	Q570
Egr1 Probe_47	GGCAATAGAGCGCATTCAAT	Q570
Egr1 Probe_48	AGGATACACACCACATATCC	Q570
MS2PP7_hybrid_1	GATGAACCCTGGAATACTGG	Q670
MS2PP7_hybrid_2	CTTGGCAATAAGTACCGTAG	Q670
MS2PP7_hybrid_3	TTTGAAGATTCGACCTGGAG	Q670

Probe sequence name	Sequence (5' to 3')	Reporter Dye
Fos Probe_1	GTTGAAACCCGAGAACATCA	Q570
Fos Probe_2	AGGATGACGCCTCGTAGTCG	Q570
Fos Probe_3	TAGTAGGAAAGGCTGTCCCC	Q570
Fos Probe_4	GAAGGAGTCGGCTGGGGAAT	Q570
Fos Probe_5	TTGACAGGAGAGCCCATGCT	Q570
Fos Probe_6	ATCTGCGCAAAAGTCCTGTG	Q570
Fos Probe_7	TAAAGTTGGCACTAGAGACG	Q570
Fos Probe_8	TGGAGATGGCTGTCACCGTG	Q570
Fos Probe_9	AGCCACTGCAGGTCTGGGCT	Q570
Fos Probe_10	GGAGACCAGAGTGGGCTGCA	Q570
Fos Probe_11	TCTGGTCTGCGATGGGGCCA	Q570
Fos Probe_12	GGAGTCCGTAAGGATGGGGC	Q570
Fos Probe_13	TAAGCCCCAGCAGACTGGGT	Q570
Fos Probe_14	TCTTCACCATTCCCGCTCTG	Q570
Fos Probe_15	TGCGCTCTGCCTCCTGACAC	Q570
Fos Probe_16	TTTGCCCCTTCTGCCGATGC	Q570
Fos Probe_17	CTTCAGGAGATAGCTGCTCT	Q570
Fos Probe_18	CGGATTCTCCGTTTCTCTTC	Q570
Fos Probe_19	AGCCATCTTATTCCGTTCCC	Q570
Fos Probe_20	TCCGATTCCGGCACTTGGCT	Q570

Fos Probe_21	AGTGTATCTGTCAGCTCCCT	Q570
Fos Probe_22	AAGTTGATCTGTCTCCGCTT	Q570
Fos Probe_23	GCAACGCAGACTTCTCATCT	Q570
Fos Probe_24	AGCAGATTGGCAATCTCAGT	Q570
Fos Probe_25	CTCCAGTTTTTCCTTCTCTT	Q570
Fos Probe_26	GTCGGTGGGCTGCCAAAATA	Q570
Fos Probe_27	TCATCGGGGATCTTGCAGGC	Q570
Fos Probe_28	CATCTCCTCTGGGAAGCCAA	Q570
Fos Probe_29	TCAAATCCAGGGAGGCCACA	Q570
Fos Probe_30	TGGAAGCCTCAGGCAGACCT	Q570
Fos Probe_31	TGAAGGCCTCCTCAGACTCT	Q570
Fos Probe_32	AGGGTCGTTGAGAAGGGGCA	Q570
Fos Probe_33	TCTTGACTGGCTCCAAGGAT	Q570
Fos Probe_34	TTCAGCTCCACGTTGCTGAT	Q570
Fos Probe_35	GAAGTCATCAAAGGGTTCTG	Q570
Fos Probe_36	GCCTAGATGATGCCGGAAAC	Q570
Fos Probe_37	ACAGAGCGGGAGGTCTCTGA	Q570
Fos Probe_38	AACCGGACAGGTCCACATCT	Q570
Fos Probe_39	TCCCAGTCTGCTGCATAGAA	Q570
Fos Probe_40	CAAGGAATTGCTGTGCAGAG	Q570
Fos Probe_41	TCTGTGACCATGGGCCCCAT	Q570
Fos Probe_42	TACAGGTGACCACGGGAGTA	Q570
Fos Probe_43	AAGACGTGTAAGTAGTGCAG	Q570
Fos Probe_44	TCAGGGTAGGTGAAGACAAA	Q570
Fos Probe_45	ACAGCTTGGGAAGGAGTCAG	Q570
Fos Probe_46	TGCTGCCCTTTCGGTGGGCA	Q570
Fos Probe_47	TCGGAGGAGGGCTCGTTGCT	Q570
Fos Probe_48	AGCAGCGTGGGTGAGCTCAG	Q570

Table S2. Average values for ON and OFF duration, Related to Figure 5.

	MCP-GFP (mean ± SEM)	PCP-GFP (mean ± SEM)	p-value
ON duration	8.3 ± 0.3	7.6 ± 0.3	0.064
OFF duration	6.9 ± 0.3	6.9 ± 0.4	0.487

(ON duration: MCP-GFP, n = 1135; PCP-GFP, n = 834. OFF duration: MCP-GFP, n = 596; PCP-GFP, n = 479)

Supplementary Figures

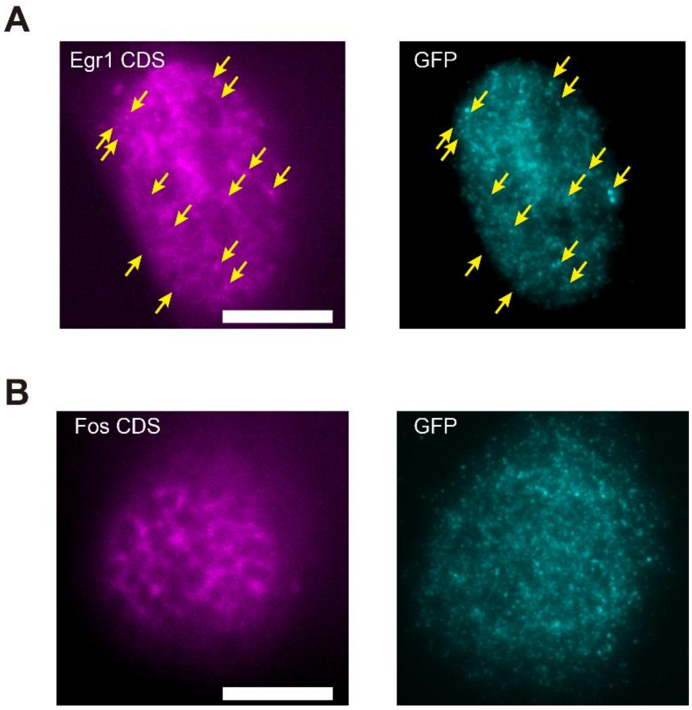

Figure S1. Validation of Egr1 and MCP-GFP/PCP-GFP colocalization, Related to Figure 2. Representative images of combined immunofluorescence (IF) for GFP and single-molecule FISH (smFISH) in immortalized MEFs. These cells were derived from the Egr1-MBPS mouse line and subsequently engineered to express both PCP-GFP and MCP-GFP. **(A)** Colocalization between the Egr1 CDS FISH probe (magenta) and the GFP signal (cyan). **(B)** Representative images showing a lack of colocalization between the Fos CDS FISH probe (magenta, negative control) and GFP signal (cyan). Scale bar, 10 μm.

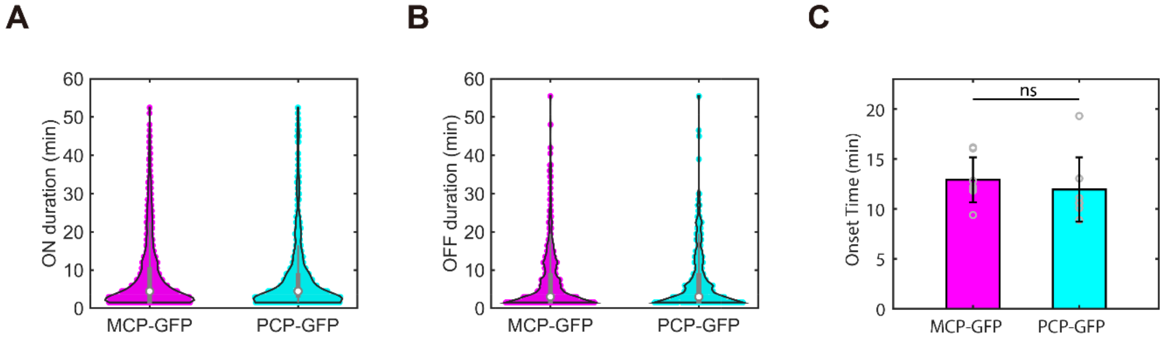

Figure S2. Distribution of transcriptional ON and OFF durations and onset times, Related to Figure 5. (A) Violin plots of ON durations for transcription sites labeled with MCP-GFP and PCP-GFP. **(B)** Violin plots of OFF durations for transcription sites labeled with MCP-GFP and PCP-GFP. Median and interquartile ranges are indicated. **(C)** Quantification of transcription onset times. Each gray dot represents the mean onset time per imaging dish, calculated by averaging onset times of all transcription sites within that dish. Bars represent mean ± SD across dishes. The average onset time was 13 ± 2 min (mean ± SD) for Egr1-MPBS × MCP and 12 ± 3 min (mean ± SD) for Egr1-MPBS × PCP. No statistically significant difference was observed between the two groups (pairwise t-test, $P > 0.05$).

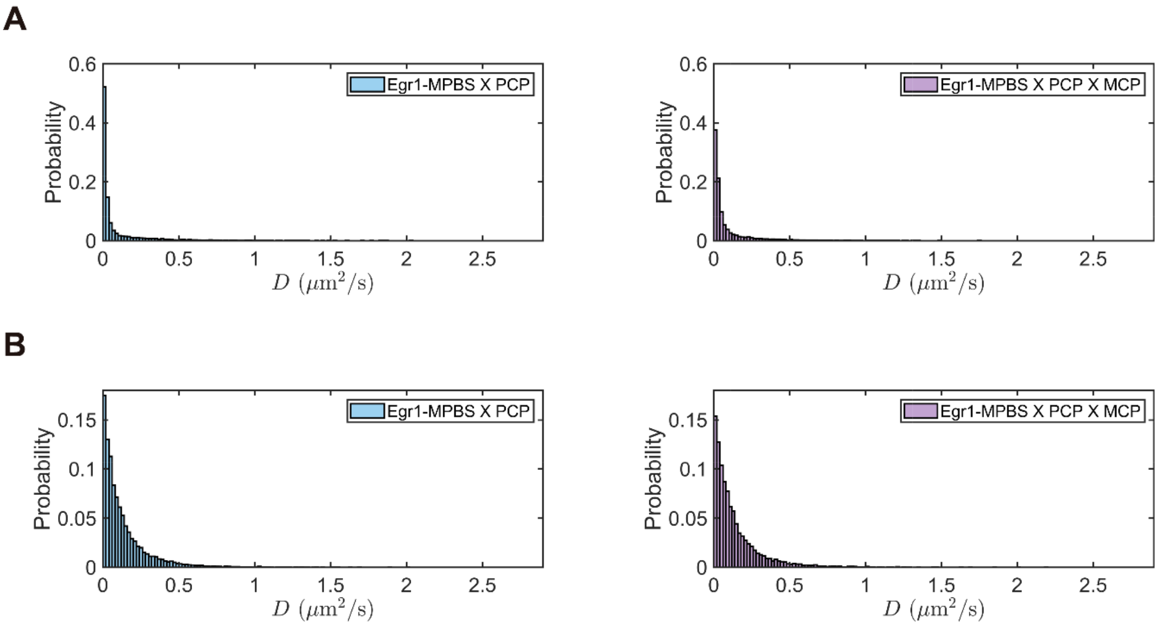

Figure S3. Distribution of mRNA diffusion coefficients in the nucleus and cytoplasm, Related to Figure 6. (A) Histogram of diffusion coefficients for nuclear Egr1 mRNAs labeled with PCP-GFP or PCP- plus MCP-GFP. **(B)** Histogram of diffusion coefficients for cytoplasmic Egr1 mRNAs labeled with PCP-GFP or PCP- plus MCP-GFP.
